# Interleukin-6 as a predictive biomarker of systemic progression and therapeutic target in cutaneous plasmacytosis^☆^

**DOI:** 10.1016/j.abd.2026.501306

**Published:** 2026-03-26

**Authors:** Yuan-Yu Hong, Peng-Yu Chen, Hui-Ting New, Sui-Qing Cai

**Affiliations:** Department of Dermatology, The Second Affiliated Hospital, Zhejiang University School of Medicine, Hangzhou, China

Dear Editor,

Cutaneous Plasmacytosis (CP), a rare disorder predominantly affecting Asian populations, is characterized by violaceous plaques with histopathological features of polyclonal mature plasma cell infiltration.^1^ Emerging data suggest systemic involvement in CP, though its incidence and treatments remain incompletely characterized.^2^ Therefore, we conducted a systematic reviews and meta-analyses (PRISMA) ‒ compliant systematic review across PubMed, Embase, Scopus and Cochrane databases (search strategy: “cutaneous or skin” and “plasmacytosis”) to clarify systemic involvement patterns and therapeutic approaches in CP.

Case reports, series and retrospective studies written in English or Chinese published before November 13, 2024, describing CP cases were included. All required pathologically confirmed prominent mature plasma cell infiltration with polyclonality, verified by κ/λ immunostaining or gene rearrangement analysis. Cases primarily involving mucosal and lymphoplasmacytic plaque in children were excluded, as they are considered separate disease entities.^3^, ^4^ We also excluded cases with possible infection-induced plasmacytosis, including active syphilis, Human Herpesvirus-8 (HHV-8), or ehrlichiosis.

Seventy-nine articles reporting 109 CP cases were identified (Fig. 1). Patients were stratified by systemic involvement: localized CP (no evidence of extracutaneous infiltration) or systemic CP (pathologically confirmed extracutaneous polyclonal plasma cell infiltration). Among 109 patients (mean age 51.0 ± 14.6 years; M:F ratio 1.6:1; 84.5% Asian), systemic involvement occurred in 37 (33.9%), primarily affecting lymph nodes (n = 23, 21.1%), bone marrow (n = 21, 19.3%), kidney (n = 4, 3.7%), lungs (n = 2, 1.8%), and ectopic masses (n = 2, 1.8%, ureteral/breast lesions), with 12 patients (11.0%) having more than one organ involved.

**Figure 1:**
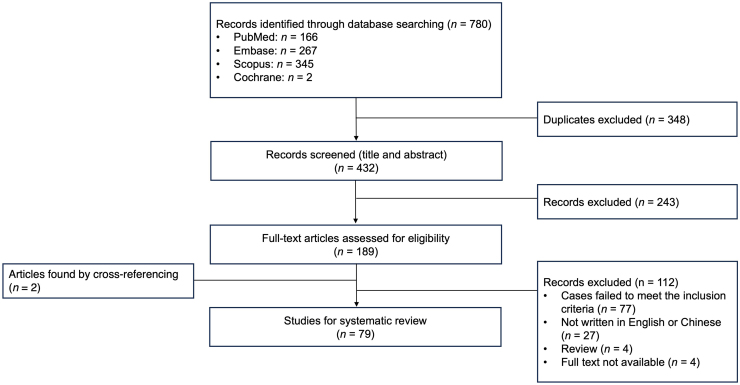
Study selection flow diagram.

We analyzed clinical characteristics, laboratory parameters, imaging findings, and pathological features documented in ≥ 40 cases to identify factors associated with systemic involvement, and compared treatment patterns and outcomes between localized and systemic CP (Table 1). Leukocytosis, polyclonal hypergammaglobulinemia, and histologically confirmed Germinal Center (GC) formation showed significant associations with systemic involvement (p < 0.05). Stronger correlations emerged for imaging detection of lymphadenopathy or hepatosplenomegaly, constitutional symptoms, anemia, and Interleukin-6 (IL-6) elevation (p < 0.001). To isolate early pathogenic drivers rather than systemic consequences, multivariate analysis focused on IL-6 and GC formation. After adjusting for demographics, duration, and GC status, IL-6 elevation emerged as the sole independent predictor (adjusted OR = 18.4, 95% CI = 2.6‒128.7, p = 0.003).

**Table 1:** Comparative demographic, laboratory, and clinical features in localized vs. systemic Cutaneous Plasmacytosis (CP).

Parameter	Localized CP (n = 72)	Systemic CP (n = 37)	p-value^a^
Male, n (%)	45 (62.5)	22 (59.5)	0.757
Age, mean (SD), years	50.5 (15.1)	52.0 (13.6)	0.613
Symptom duration before diagnosis, median (IQR), months	42.0 (72.0)	48.0 (57.0)	0.550
Imaging detection of lymphadenopathy or hepatosplenomegaly, n (%)	7 (9.7)^b^	18 (48.6)	<0.001
Constitutional symptoms, n (%)	1 (1.4)	9 (24.3)	<0.001
Anemia, n/N (%)	6/36 (16.7)	15/17 (88.2)	<0.001
Leukocytosis, n/N (%)	3/34 (8.8)	5/11 (45.5)	0.014
Polyclonal hypergammaglobulinemia, n/N (%)	27/44 (61.4)	28/32 (87.5)	0.012
Elevated IgG, n/N (%)	38/45 (84.4)	25/27 (92.6)	0.520
Elevated IgA, n/N (%)	21/31 (67.7)	13/18 (72.2)	0.743
Elevated IgM, n/N (%)	13/27 (48.1)	11/20 (55.0)	0.642
Elevated serum IL-6, n/N (%)	8/24 (33.3)	15/17 (88.2)	<0.001
LF/LF-like structure present, n (%)	16 (22.2)	13 (35.1)	0.149
LF with germinal center, n (%)	9 (12.5)	11 (29.7)	0.028
Systemic therapy received, n/N (%)	27/51 (52.9)	18/25 (72.0)	0.112
Treatment response rate, n/N (%)	32/44 (72.7)	15/22 (68.2)	0.701
Adverse outcomes^c^, n/N (%)	6/19 (31.6)	4/14 (28.6)	1.00

CP, Cutaneous Plasmacytosis; SD, Standard Deviation; IQR, Interquartile Range; IL-6, Interleukin-6; LF, Lymphoid Follicle.

a Quantitative variables were analyzed using *t*-tests or Mann-Whitney tests; categorical variables with χ² or Fisher's exact tests, as appropriate.

b Among 7-localized CP cases with imaging-detected lymphadenopathy or hepatosplenomegaly, 6-lacked confirmatory biopsies.

c Adverse outcomes are defined as progressive diseases, development of malignancies, or all-cause mortality.

Treatment data existed for 76 patients (69.7%), with outcomes evaluable in 66 (60.5%) (Table 2). We assigned patients receiving sequential therapies to separate treatment groups based on clinical responses, excluding therapeutic approaches documented in ≤ 2 patients. Topical therapies were corticosteroids (Overall Response Rate [ORR] = 50%) and calcineurin inhibitors (ORR = 60%), primarily for localized CP. Systemic corticosteroids served as the mainstay for both localized CP (n = 8, ORR = 75%) and systemic CP (n = 8, ORR = 100%), though most achieved only partial response with potential relapse after tapering. Immunosuppressant add-ons provided no ORR improvement. The CHOP chemotherapy regimen (cyclophosphamide, doxorubicin, vincristine, and prednisone) showed efficacy in 3 refractory cases (ORR = 100%). PUVA (psoralen-UVA therapy) and NB-UVB (narrowband-ultraviolet B) were primarily used in patients with localized CP, and demonstrated ORR of 50% and 40%, respectively. Six patients received IL-6 pathway-targeting inhibitors. One localized CP patient on unspecified anti-IL-6 antibody showed no response, 2 systemic CP patients on siltuximab demonstrated no efficacy, and the outcome was unreported for each patient, respectively.^5^, ^6^, ^7^ Notably, 3 tocilizumab-treated systemic CP patients achieved 100% ORR (1 with partial improvement, 2 with significant rash reduction).^8^, ^9^, ^10^ Conversely, all 3 rituximab (CD20 inhibitor)-treated systemic CP patients exhibited no therapeutic effects.

**Table 2:** Treatment regimens and response rates in Cutaneous Plasmacytosis (CP).

Treatment Category	Regimen	Localized CP (n = 51)		Systemic CP (n = 25)	
		Cases	ORR (%)^a^	Cases	ORR (%)
Topical Therapies					
Corticosteroids	Monotherapy	8	50	‒	‒
Calcineurin inhibitors	Monotherapy	5	60	‒	‒
Systemic Agents					
Corticosteroids	Monotherapy	8	75	8	100
	Combination^b^	10	50	4	75
Chemotherapy	CHOP	1	100	2	100
Physical Therapies					
PUVA	All regimens^c^	8	50	1	0
NB-UVB	All regimens	5	40	‒	‒
Biologics					
Anti-IL-6	Tocilizumab	‒	‒	3	100
	Siltuximab	‒	‒	1	0
	Unspecified	1	0	‒	‒
Anti-CD20	Rituximab	‒	‒	3	0

CP, Cutaneous Plasmacytosis; ORR, Overall Response Rate; CHOP, Cyclophosphamide, Doxorubicin, Vincristine, and Prednisone; PUVA, Psoralen-UVA therapy; NB-UVB, Narrowband Ultraviolet B; IL-6, Interleukin-6.

a ORR: overall response rate (%) = [(complete response + partial response) cases / total evaluable cases] × 100%.

b Combination refers to the concurrent use of immunosuppressants such as methotrexate, cyclophosphamide, or thalidomide with systemic corticosteroids.

c All regimens comprise all therapeutic approaches for PUVA/NB-UVB, including monotherapy and combination therapies (e.g., with topical corticosteroids).

Follow-up data existed for 36 patients (33.0%), including outcomes for 33 (30.3%). Median follow-up was 27.0 ± 48.0 months. Adverse outcomes occurred in 6/19 (31.6%) localized CP patients: progressive disease, fatal respiratory and cardiac failure, T-cell lymphoma, and Castleman disease. Among 14 systemic CP patients, 4 (28.6%) had adverse outcomes: progressive disease with elevated IL-6, sudden death, acute myeloid leukemia M2, and gastric adenocarcinoma. Two tocilizumab-treated patients maintained complete remission at 10- and 18-months.^9^, ^10^

IL-6, a pleiotropic cytokine driving plasma cell differentiation and inflammatory cascades, is identified as a primary biomarker of systemic progression in CP, supporting its utility for early risk stratification. Although our study showed no prognostic difference between localized and systemic CP, this may be obscured by the retrospective study design, limited sample size, and incomplete patient assessment and biomarker documentation. Tocilizumab, an IL-6 receptor blocker, demonstrates superior efficacy to direct IL-6 inhibitors, representing a promising therapeutic candidate. Future multicenter prospective studies employing standardized diagnostic frameworks and biomarker-driven stratification are needed to validate these findings and establish evidence-based guidelines.

## ORCID ID

Yuan-Yu Hong: 0009-0004-6638-4569

Peng-Yu Chen: 0000-0002-1041-4130

Hui-Ting New: 0009-0002-9455-1729

Sui-Qing Cai: 0000-0002-7543-9013

## PROSPERO registration number

CRD42025649464.

## Research data availability

The entire dataset supporting the results of this study was published in this article.

## Financial support

None declared.

## Authors' contributions

Yuan-Yu Hong: Critical literature review; data collection, analysis and interpretation; preparation and writing of the manuscript; statistical analysis; study conception and planning.

Peng-Yu Chen: Data collection, analysis and interpretation; preparation and writing of the manuscript; statistical analysis; study conception and planning.

Hui-Ting New: Data collection, analysis and interpretation; manuscript critical review.

Sui-Qing Cai: Approval of the final version of the manuscript.

## Conflicts of interest

None declared.

## References

[1] Wagner G., Rose C., Klapper W., Sachse M.M. (2013). Cutaneous and systemic plasmocytosis. J Dtsch Dermatol Ges..

[2] Watanabe S., Ohara K., Kukita A., Mori S. (1986). Systemic plasmacytosis. a syndrome of peculiar multiple skin eruptions, generalized lymphadenopathy, and polyclonal hypergammaglobulinemia. Arch Dermatol..

[3] Mitteldorf C., Palmedo G., Kutzner H., Kauer F., Prestin M., Schuster C. (2015). Diagnostic approach in lymphoplasmacytic plaque. J Eur Acad Dermatol Venereol..

[4] Smith M.E., Crighton A.J., Chisholm D.M., Mountain R.E. (1999). Plasma cell mucositis: a review and case report. J Oral Pathol Med..

[5] Haque M., Hou J.S., Hisamichi K., Tamada K., Cusack C.A., Abdelmalek M. (2011). Cutaneous and systemic plasmacytosis vs. cutaneous plasmacytic Castleman disease: review and speculations about pathogenesis. Clin Lymphoma Myeloma Leuk..

[6] Gutierrez R.A., Jacobson R., Dawson A., Haemel A., North J. (2023). Cutaneous and systemic plasmacytosis with concurrent lymphoid hyperplasia. J Cutan Pathol..

[7] Drissi M., Dunlap R., Clayton L., Raess P.W., Mengden Koon S., White K. (2022). Cutaneous plasmacytosis and idiopathic multicentric Castleman disease: a spectrum of disease?. Am J Dermatopathol..

[8] Almazan T., Jung J. (2016). Cutaneous plasmacytosis treated with tocilizumab: a case report and review of the literature. Madridge J Dermatol Res..

[9] Aita T., Hamaguchi S., Shimotani Y., Nakamoto Y. (2020). Idiopathic multicentric Castleman disease preceded by cutaneous plasma cytosis successfully treated by tocilizumab. BMJ Case Rep..

[10] Daftary K., Figueredo Zamora E., Malone J., Callen J.P. (2024). Cutaneous plasmacytosis treated with tocilizumab. Clin Exp Dermatol..

